# Hypoxia in Cell Reprogramming and the Epigenetic Regulations

**DOI:** 10.3389/fcell.2021.609984

**Published:** 2021-01-28

**Authors:** Nariaki Nakamura, Xiaobing Shi, Radbod Darabi, Yong Li

**Affiliations:** ^1^Department of Orthopaedic Surgery, and Biomedical Engineering, Homer Stryker M.D. School of Medicine, Western Michigan University, Kalamazoo, MI, United States; ^2^Center for Epigenetics, Van Andel Research Institute, Grand Rapids, MI, United States; ^3^The Center for Stem Cell and Regenerative Medicine (CSCRM), Brown Foundation Institute of Molecular Medicine for the Prevention of Human Diseases (IMM), Houston, TX, United States

**Keywords:** hypoxia, epigenetic, muscle, cellular reprogramming, stem cells

## Abstract

Cellular reprogramming is a fundamental topic in the research of stem cells and molecular biology. It is widely investigated and its understanding is crucial for learning about different aspects of development such as cell proliferation, determination of cell fate and stem cell renewal. Other factors involved during development include hypoxia and epigenetics, which play major roles in the development of tissues and organs. This review will discuss the involvement of hypoxia and epigenetics in the regulation of cellular reprogramming and how interplay between each factor can contribute to different cellular functions as well as tissue regeneration.

## Introduction

When Takahashi and Yamanaka were able to reprogram a fully differentiated skin cell into an induced pluripotent stem cell (iPSC), the promising potential of cellular reprogramming became evident. Through activation of critical transcription factors Oct4, Sox2, Klf4, and c-Myc (Yamanaka factors), any somatic cell could be reprogrammed into a pluripotent stem cell (Takahashi and Yamanaka, 2006; Takahashi et al., 2007). The implications of this discovery are already applied to the development of therapies targeting macular degeneration, spinal cord injuries, acute myeloid leukemia, and more (Chichagova et al., 2018; Maali et al., 2018; Nagoshi et al., 2019). However, the initial method of cellular reprogramming because <1% of the cells committed to reprogramming actually gained pluripotency (Takahashi et al., 2007). This inefficiency necessitates that better methods of cellular reprogramming be developed for practical use.

One such method is the culturing of cells in hypoxic conditions. In early development, before the formation of the placenta, the developing embryo is exposed to an environment that varies between 1 and 5% O_2_ a concentration much lower than the physiologically normal 20–21% O_2_ (Yoshida et al., 2009). When the developing embryo reaches the blastocyst stage, embryonic stem cells (ESCs) are extracted from an inner cell mass (ICM) contained within an outer layer of cells (Thomson et al., 1998; Schrode et al., 2013). ESCs are pluripotent and display high expression of the original Yamanaka factors as well as other transcription factors: Nanog and Lin28A (Moss and Tang, 2003; Pan and Thomson, 2007; Yu et al., 2007).

In an experiment done to test the effect of hypoxia on the production of induced pluripotent stem cells (iPSCs), both mouse embryonic fibroblasts and human dermal fibroblasts were transduced with Yamanaka factors and cultured under normoxia (21% O_2_) or hypoxia (5% O_2_). Not only did the hypoxia treated cells grow faster, production of iPSCs significantly increased when cells were cultured under 5% O_2_ at all periods of induction (Yoshida et al., 2009). In another research, a low oxygen tension (5% O_2_) preferentially resulted in the maintenance of a highly proliferative, pluripotent population of human ESCs; Moreover, the culture at atmospheric 20% O2 levels expressed significantly less OCT4, SOX2, and NANOG than those maintained at 5% O2 in the hESCs (Forristal et al., 2010).

## Hypoxia and its Role in Gene Transcription

The initiation of hypoxia results in an upregulation of transcription factors called Hypoxia-Inducible Factors (HIFs). HIFs are activated in response certain environmental conditions, including a low oxygen environment and in inflammation (Lee et al., 2020). HIFs are composed of multiple transcription factors: HIF-1, HIF-2, and HIF-3.

HIF-1α is the main driver of the cellular response in low oxygen environments. At normal oxygen levels (20–21% O_2_), enzymes called prolyl-hydroxylases (PHDs) hydroxylate HIF-1α at proline residues 402 and 564. This modification tags HIF-1α for ubiquitination by the E3 ubiquitin ligase complex, which in turn tags it for degradation by the proteasome (Koivunen and Kietzmann, 2018). During hypoxia, the oxygen that is required for hydroxylation, the initial step of degradation, is not present and therefore HIF-1α is able to remain intact and functional. HIF-1α accumulates within the cell and dimerizes with the HIF-1β subunit forming a heterodimeric HIF-1α/HIF-1β complex. Afterwards, the complex translocates to the nucleus, where it binds to hypoxia response elements (HREs) and upregulates the expression of its target genes (Sadaghianloo et al., 2020). HIF-1α has been implicated in the preservation and adjustment of the initial physiological response to hypoxia and plays a vital role in the body's viability in situations where the oxygen level is low. Examples of such adjustments include increasing angiogenesis and glycolysis while decreasing cell proliferation (Majmundar et al., 2010). The HIF-2 mechanism of action works similarly to HIF-1. HIF-2α is unstable at physiologically normal (20%) oxygen levels because of hydroxylation at their proline residues. During hypoxia, HIF-2α dimerizes with HIF-1β and translocates to target genes in the nucleus and upregulates the expression of target genes. The difference between HIF-1 and HIF-2 lies in their target genes (Carroll and Ashcroft, 2006). HIF-2α binds to HREs upstream of cellular reprogramming genes Oct4, Sox2, Nanog, and significantly increases their expression (Covello et al., 2006; Forristal et al., 2010). HIF-2α has also been shown to upregulate c-Myc expression by modulating its interactions with cell cycle proteins and is associated with the upregulation of Klf4 (Gordan et al., 2007).

There are many studies indicating that hypoxia regulates gene transcription and expression mostly through the HIF signaling pathways. Hypoxia stimulates the transcription of genes that help to restore oxygen levels or energy productions, in which HIF responds and regulates gene transcription. Hypoxia as an environmental factor can increase muscle stem cells (MuSCs) renewal through its transcription factor HIF-1α and the Notch pathways (Liu et al., 2012; Yang et al., 2017). There is increasing evidence that hypoxia causes reprogramming of other cells, such as dendritic cells, neural cells, and muscle cells (Bosco and Varesio, 2012; Mathieu et al., 2013; Vojnits et al., 2015). The HIF1α pathway is discovered as an enabling regulator of cellular reprogramming through early glycolytic shift and upregulation of PDK1-3 and PKM2. In fact, HIF1 controls the transcription of many target genes to initiate metabolic changes in the early stage and maintains glycolytic metabolism in the later phase of the iPSC reprogramming. Hypoxia is known to enhance the efficiency of reprogramming in the development of iPSCs by upregulating the expression of transcription factors associated with cellular reprogramming and changing the topography of chromatin (Yoshida et al., 2009; Bosco and Varesio, 2012; Mimura et al., 2012; Mathieu et al., 2014; Wang et al., 2016; Alderman et al., 2019).

## Hypoxia and its Role in Development

There is a large amount of evidence that hypoxia and HIF play a role in pre-embryonic development (Dunwoodie, 2009). In the developing placenta, the knockdown of HIF-1α and HIF-2α results in failure of its formation. Trophoblast invasion, one of essential stages for placentation and pregnancy outcome, likely occurs in a hypoxic environment. Studies have indicated the hypoxia is able to induce 10–11 translocation methylcytosine dioxygenase 1 (TET1) expression that facilitates trophoblast cell migration and invasion through HIF-1α signaling pathways in the early pregnancy (Koklanaris et al., 2006; Zhu et al., 2017).

More specifically, knockdown of HIF-1α prevents the fusion of the chorion and the allantois which normally fuse together to form the placenta. Since the chorion and the allantois are not harmed by the knockdown of HIFs, this suggests that HIF plays a role in the integrins that regulate this formation. When exposed to hypoxia, the expression of some of these integrins increased (Dunwoodie, 2009). Additional research suggests that the lack of oxygen and the subsequent upregulation of HIFs plays a vital role in branching morphogenesis, the process that lays down the foundation for the development of key organs such as the nervous system, the respiratory system, the kidney, the salivary glands and the mammary glands (Dunwoodie, 2009; Tsuji et al., 2014). When HIF is knocked down, vascularization of the labyrinthine layer of the placenta decreases, preventing nutrients from the maternal circulatory system to reach the fetal one (Dunwoodie, 2009).

Further along in placental development, HIF is also involved in the formation of the trophoblast (Dunwoodie, 2009). The trophoblast is the outer layer of the developing blastocyst and plays a key role in nourishing the embryo with nutrients. During pre-embryonic development, the trophoblast is attached to the uterine lining and trophoblast cells physically bordering the lining express high amounts of HIF-1α and HIF-2α. When both subunits are knocked out, the formation of trophoblast subgroups within the trophoblast are inhibited, suggesting that HIF plays a vital role in trophoblast proliferation. As the embryo starts to form into a fetus, hypoxia again plays a major role in the development of major organs. Recent research suggests that HIF also interacts with the Notch signaling pathway. During hypoxia, the HIF complex can attach itself to a Notch receptor and induce notch signaling (Hu et al., 2014). Notch signaling is implicated in the determination of cell fate and HIF induced notch signaling has proven to be involved in determining blood cell fate as well as determining neural cell fate. Lastly, hypoxia and its corresponding HIFs have shown to play a major role in bone formation, chondrogenesis, heart formation, angiogenesis and formation of the neural crest (Rankin et al., 2011; Lee et al., 2013; Muz et al., 2015; Scully et al., 2016). Overall, HIFs play a significant part in promoting cell proliferation and differentiation of multiple cells during the development of organisms.

## Hypoxia and its Role in Disease

Other than development, hypoxia may play a role in other physiological events characterized by rapid proliferation and differentiation. Many have theorized of a link between the regulation of ESCs and the regulation of cancer stem cells (CSCs). In cancers, the rapid outgrowth of cells and their consumption of oxygen overruns the limited supply of oxygen and creates a hypoxic environment in many areas of the tumor (Muz et al., 2015). The resulting increase in HIFs plays an integral role in cancer pathogenesis by upregulating various transcription factors involved in angiogenesis and cell proliferation (Muz et al., 2015). Some state that this hypoxic environment is also favorable for the cellular reprogramming of non-stem like cancer cells into cancer-stem cells. Heddleston et al. transduced HIF-2α into non-stem like glioma cells and discovered that the expression of Oct4, Nanog, and c-Myc increased in HIF-2α transduced cells (Heddleston et al., 2009). The subsequent injection of these cells into immunocompromised mice created significantly larger tumors compared to the control. Further research supports the notion that the chemoresistance of gliomas is also a result of hypoxia induced cellular reprogramming. In tests comparing the sensitivity of glioma associated cells to chemotherapy, adult non-stem like glioma cells showed sensitivity to treatment while glioma stem cells (GSCs) remained unaffected (Wang et al., 2017a). The origin of the GSCs have been debated as some believed that GSCs were derived through contamination from cells already positive for CD133 (GSC marker) while others believed they were a result of hypoxia induced reprogramming of already present CD133^−^ glioma cells. In experiments conducted by Wang et al., they provide evidence it is the latter (Wang et al., 2017b). When CD133^−^ glioma cells were cultured under hypoxia, expression of CD133 increased and signs of GSC behavior such as the ability to asymmetrically divide and the ability to form neurospheres appeared. Hypoxia cultured cells also showed increased expression of ABCG2 and MGMT, proteins associated with increased chemoresistance.

The presence of cells with stem-like properties after hypoxia is not confined to gliomas. Stem-like cells induced from hypoxia have been discovered in cells associated with lung cancer and liver cancer. In non-oncologic fields, increasing amounts of evidence suggest that hypoxia induces the reprogramming of resident muscle cells after injury. Novel multipotent cells have been discovered in the tibialis anterior (TA) muscle in mice after a laceration injury disrupted the vascular structure (Mu et al., 2011). These cells were termed as injury-induced muscle-derived stem cells or iMuSCs and have shown the ability to differentiate into cells from all three germ layers and form neurospheres (Vojnits et al., 2015, 2017). Similar cells, termed ischemia-induced multipotent stem cells or iSCs, have also been discovered in the brain of elderly patients after an ischemic stroke (Tatebayashi et al., 2017). The multipotent or pluripotent nature of iMuSCs and iSCs and their presence in tissues after injury point to the therapeutic potential of hypoxia induced cellular reprogramming in muscle and neural regeneration. However, like the aforementioned GSCs, some may argue that the presence of iMuSCs and iSCs can be the result of contamination from circulating cells. *In vitro* studies that can replicate the formation of such cells through hypoxia or studies identifying the mechanism behind their reprogramming will be useful in clarifying their roles.

### Epigenetics in SCNT and iPSCs Reprogramming

Another major factor involved in the development of stem cells is epigenetics. Epigenetics refers to the cellular machinery that controls gene expression and occurs through the addition or deletion of epigenetic modifications (Handy et al., 2011). Epigenetic modifications refer to any heritable modifications that are made to the DNA or histones. The most basic of these are DNA and histone methylation, but other forms of modification can occur in histones such as acetylation, phosphorylation, ubiquitination, sumoylation etc. These modifications alter the accessibility of chromatin to transcription factors and polymerases that play a role in the transcription of DNA and expression of genes (Handy et al., 2011). Epigenetic modifications play major roles in development and in the development of disease (Portela and Esteller, 2010).

DNA methylation in general exists as a suppression mechanism for many genes. As organisms develop from the embryo into a fetus, areas in the genome with large amounts of CG dinucleotide repeats, known as CpG islands, experience methylation on the cytosine residue (Portela and Esteller, 2010; Handy et al., 2011). This hypothetically serves to suppress pluripotency at critical genes as pluripotent embryonic stem cells differentiate into their preferred cells and organ systems. The methyl groups block transcription by blocking the attachment of transcription factors onto the DNA segment. Interestingly, methyl groups have also shown to block HIF-1 from attaching to its HRE to regulate erythropoietin transcription (Handy et al., 2011). In addition to blocking transcription factors and polymerases, methyl groups are targeted by MeCP2 proteins, which recruit histone deacetylases (HDACs) to condense the chromatin and prevent transcription (Portela and Esteller, 2010; Handy et al., 2011).

Histones modifications have wide-ranging effects. Histones serve as molecular chaperones and organize DNA into structures called nucleosomes, which consist of 5 subunits, H1, H2A, H2B, H3, and H4. The subunits H2A, H2B, H3, and H4 consist of the core proteins and are bound together to form “beads” in which DNA strands wrap around. H1 serves as the support that keeps the DNA strands and the core histones in place. The number of histone subunits relates directly to the diversity of epigenetic modifications found in histones. Each core histone subunit has a multitude of modifications the most common of which are found in H3 (Handy et al., 2011). H3 modifications are widely studied and have a significant impact on the activation and repression of genes. The nomenclature of these modifications proceeds in the following order: the subunit of the histone, the amino acid affected, the position of the amino acid, and the type of modification applied. For instance, H3K27me3 indicates a trimethylation at H3 on Lysine 27. Some commonly observed modifications on H3 are H3K4me1, H4K4me3, H3K36me3, H3K79me2, H3K9Ac, H3K27Ac, all of which are associated with the opening up of the chromatin structure and gene activation by transcription factors and polymerase. In contract, H3K9me3 and H3K27me3 are associated with the condensation of chromatin and the blocking of gene expression. It has been found that H3K27me3 imprinting defects impede post-implantation development (Matoba et al., 2018). H3K9me3 and H3K4me3 might also affect transcriptional reprogramming and thus impair the developmental potential of SCNT embryos (Matoba and Zhang, 2018). H3K9me3 also showed to be implicated in the majority of barriers to the Somatic Cell Nuclear Transfer (SCNT) and iPSCs reprogramming. Those data suggest the essential, diverse set of roles of epigenetics in cellular reprogramming (Wang et al., 2018).

### Epigenetic Modifications in Stem Cell Potency

**Figure 1:**
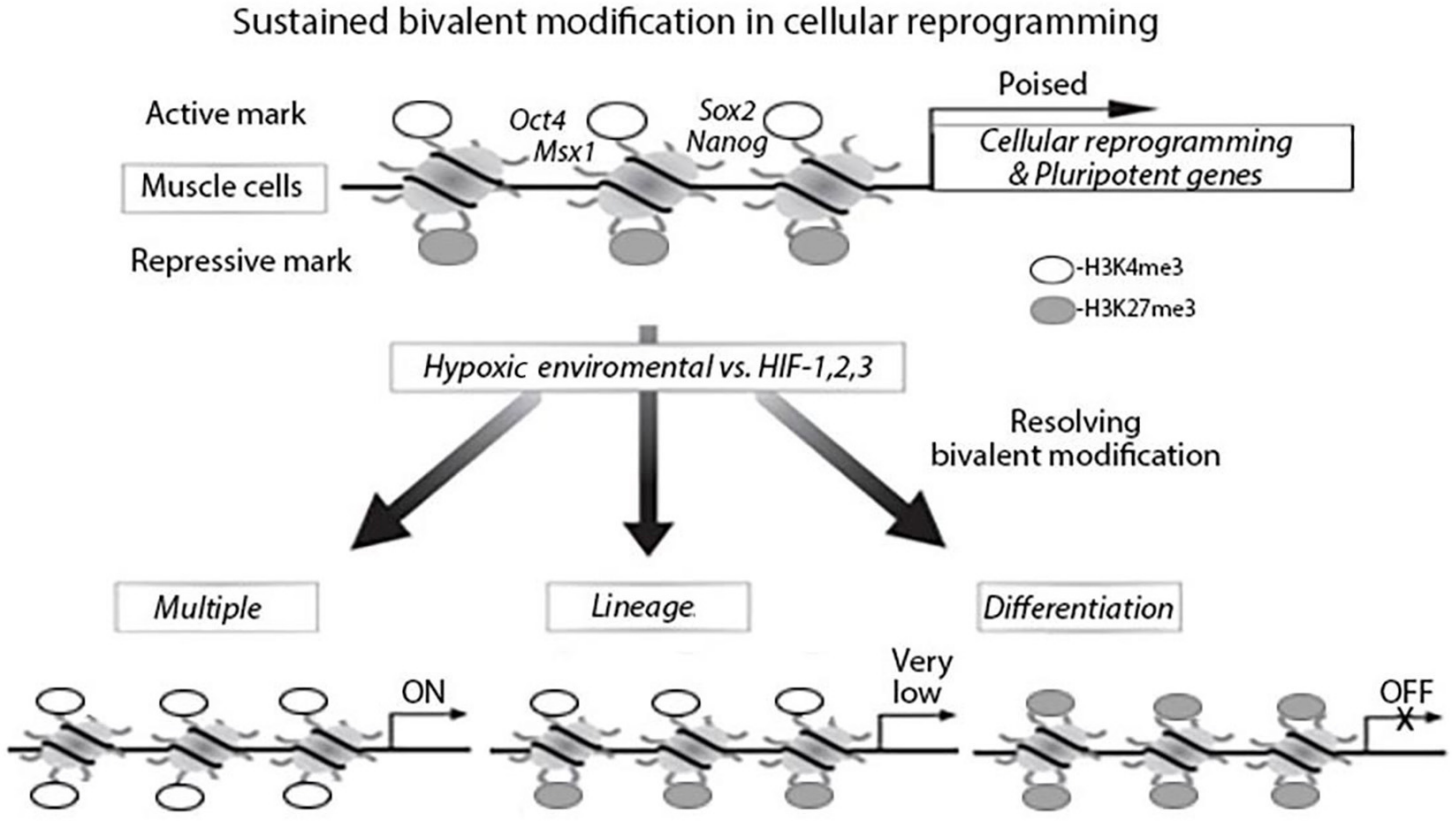
Bivalent modification status of genes in muscle cells in hypoxia environment, myogenetic differentiation and cellular reprogramming. In muscle cells, cellular reprograming genes and pluripotent genes are epigenetically modified with bivalent modifications (H3K4me3 and H3K27me3) and are poised for expression. Under hypoxia environment, the bivalent modifications are resolved; genes with H3K27me3 remained are silenced and genes with H3K4me3 remained are actively expressed.

Histone modifications are involved in development to set up genes for activation during lineage commitment by H3K4me3 and to repress lineage control genes to maintain pluripotency by H3K27me3 (Shipony et al., 2014). The balance and interaction between these pathways are essential for stem cell homeostasis and are directly linked to cellular behaviors. H3K27me3 and PRC2 each contribute to epigenetically transmitting the memory of repression across generations and during development (Juan et al., 2011; Stojic et al., 2011). H3K27me3 and H3K4me3 promoter bivalency are observed in stem cells and their differentiation, which include embryonic and iPSCs (Liu et al., 2013, 2016; Leschik et al., 2015). Bivalency has a prominent role in post-implantation embryonic development or post-natal organ growth. However, the role of epigenetic regulation in the function of adult tissue stem cells is less well-understood. It has been discovered H3K27ac (acetylation) regulates cell reprogramming and mouse ES cell differentiation as well as MyoD expression (Mattout et al., 2011; Khilji et al., 2018; Martire et al., 2019). The effects of epigenetic dysregulation on adult stem cell function depend on the tissue type and the epigenetic regulator affected. Recently studies have increased our understanding of the epigenetic regulations during MuSCs maintenance, activation, differentiation, and homeostasis (Figure 1) (Liu et al., 2013; Kosan et al., 2018).

In the studies of bone marrow mesenchymal stem cells (MSCs), results indicate that hypoxia could induce senescence of MSCs via altered gut microbiota (Xing et al., 2018). However, most reports suggested that hypoxia inhibits senescence of MSCs and maintains their properties (Tsai et al., 2011; Kwon et al., 2017; Korski et al., 2019). In the cardiovascular system, hypoxia involves resident cell senescence either via promotion or prevention of the processes, suggesting the signaling pathways are complex (Korski et al., 2019; Lewinska et al., 2020). It also suggests that the level of O2 may determine the final role of hypoxic environment. Epigenetic events are essential to establish and maintain the distinct cell lineages and have been shown to be crucial players controlling the fate and function of MSCs and MuSCs(Nakade et al., 2017; Zhang et al., 2019). More recently, a study showed MuSCs forced to express Msx1 exhibited increased proliferation index as well as promoted SSEA1 and Pax7 expression, but restricted MyoD expression and accessed osteogenic genes (Ding et al., 2017).

## Hypoxia Impacts Histone Modification and Epigenetic Regulation

The Polycomb repressive complex 2 or PRC2 is a histone methyltransferase complex and functions as a transcriptional corepressor (Lee et al., 2006). PRC2 consists of three subunits, SUZ12, EED, and EZH2. EZH2 is the catalytic subunit of PRC2 that can mono-, di-, and trimethylate H3K27 (Conway et al., 2015). In ESCs, PRC2 has been shown to occupy genes that are essential for differentiation and development, and represses those genes by depositing the repressive H3K27me3 mark. When cells undergo differentiation, these genes exhibit a loss of PRC2 occupancy and a loss H3K27 trimethylation. Depletion of PRC2 or its associated subunits in ESCs or mice results in the disappearance of pluripotency, the displacement of H3K27me3, and premature differentiation during early development (Koppens et al., 2016; Shan et al., 2017). PRC2 KO mice are non-viable and die early in development (Pasini et al., 2004). These results suggest that PRC2 plays a crucial role during early development by repressing select developmental genes and maintaining ESC pluripotency.

**Figure 2:**
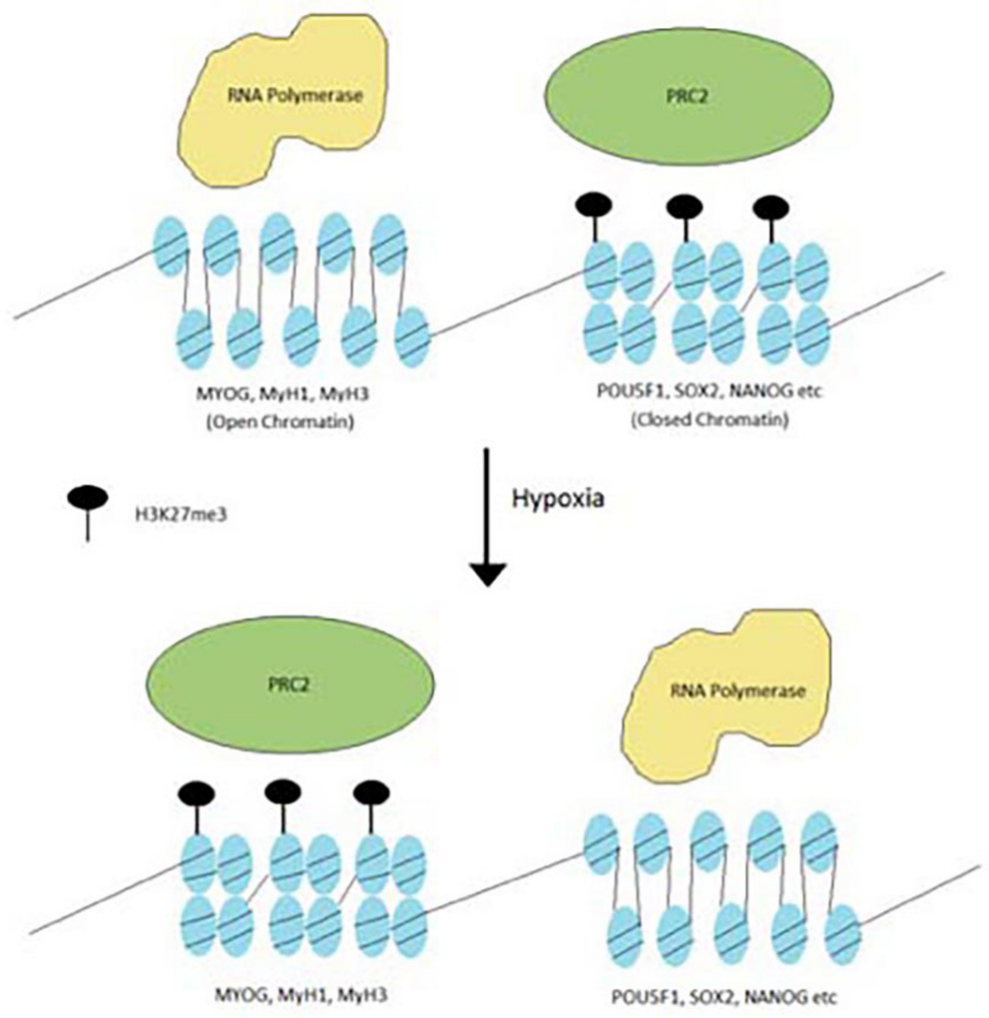
Redistribution of H3K27me3 under hypoxia. Hypoxia stimulates the PRC2 complex to redistribute H3K27me3 from oncogenic genes to differentiation genes.

PRC2 subunits are frequently mutated in cancers (Lee et al., 2006; Conway et al., 2015; Laugesen et al., 2016; Veneti et al., 2017). These mutations are observed in a variety of cancers such as breast cancer, colorectal cancer, hepatocellular carcinoma and glioblastoma. Most of the PRC2 mutations are inactivation mutations, leading to downregulation of H3K27me3 levels. While H3K27me3 is downregulated, it is never fully eliminated and in fact may appear in greater concentrations in select areas of the genome. In some other cancers, such as lymphomas, EZH2 has hot-spot gain of function mutations. In this case, levels of H3K27me3 actually increased and differentiation is blocked. This points to the idea that oncogenic PRC2 mutations redistribute H3K27me3 from genes that cause cancer and redistribute them toward genes that control differentiation (Figure 2) (Conway et al., 2015).

Another histone methylation notable for its involvement in pluripotency is H3K4me3. H3K4me3 is an active mark enriched at gene promoters. There is a set of genes that contain both H3K4me3 and H3K27me3 on their promoters, known as bivalent domains (Bernstein et al., 2006). The presence of both H3K4me3 and H3K37me3 at the developmental regulator genes puts these genes under bivalent epigenetic control. When ESCs undergo differentiation, H3K27me3 disappear from the region and is increasingly concentrated with the activating H3K4me3. On a fully differentiated gene, its promoter is only populated with H3K4me3. Downregulation of H3K27me3 via EZH2 knockout in ESCs or muscle stem cells forces the cell to undergo differentiation (Yu et al., 2018; Wang et al., 2019). These findings suggest that bivalent chromatin plays a critical role in the maintenance of stem cell pluripotency and regulation of stem cell differentiation. There are multiple interactions of epigenetic regulations during cellular reprogramming of muscle stem cells in a hypoxia environment. Hypoxia vs. active HIF-1α can increase genome-wide bivalent epigenetic making that could be an initial stimulator in cellular reprogramming. These epigenetic modifications and chromatin states may not only regulate cellular reprogramming but also normalize the multiple differentiation in a different way to support muscle regeneration (Liu et al., 2013; Faralli et al., 2016).

### Hypoxia Influences Epigenetics Modifications in Cellular Reprogramming

Recent studies have shown that hypoxia can affect the level of epigenetic modifications in cells. In breast epithelial adenocarcinoma MCF7 cells, hypoxia increased the levels of H3K27me3 globally and increased the number of genes under bivalent epigenetic control by increasing levels of H3K4me3 in H3K27me3 concentrated areas (Prickaerts et al., 2016). When reoxygenated, many of the genes retained the bivalency and conferred an epigenetic profile similar to those of embryonic stem cells. Activity of the H3K27me3 demethylase KDM6B/JMJD3 decreased as well, indicating hypoxic control of H3K27me3 demethylation (Prickaerts et al., 2016). In HeLa cells and human fibroblasts, hypoxia increases the levels of H3K4me3 in enhancers of genes associated with cell division and oxidative phosphorylation (Batie et al., 2019). The increase in histone marks at select genes associated with cell proliferation and differentiation matches favorably with the increase in transcription factors, Oct4, Sox2, and Nanog associated with cellular reprogramming. In fact, the addition of Oct4, Sox2, and Nanog to mesenchymal stem cells results in an increase in cell proliferation (Han et al., 2014; Park et al., 2019). A similar increase in cell proliferation was seen when treating mesenchymal stem cells under hypoxia (Kwon et al., 2017).

**Figure 3:**
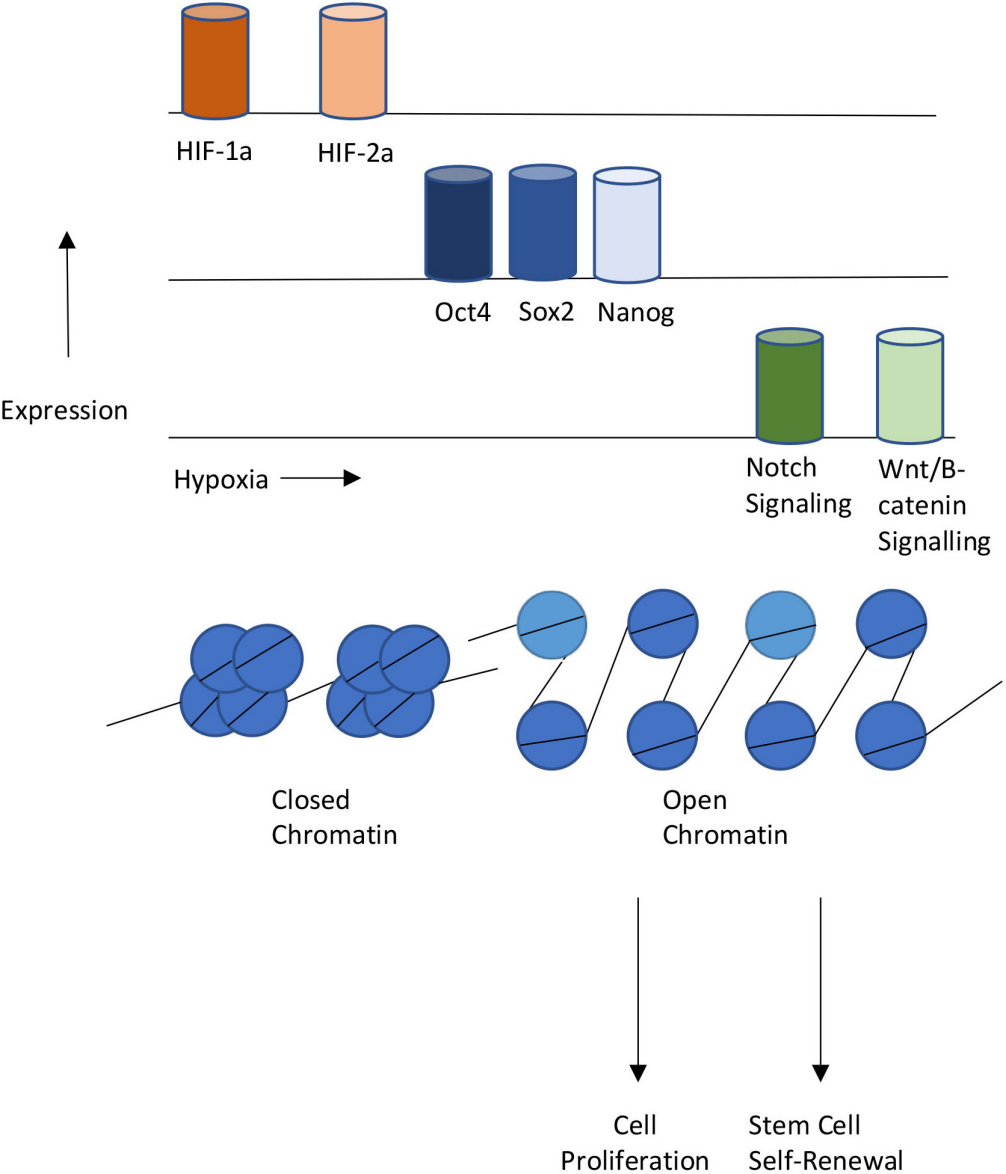
Interplays between Hypoxia, Epigenetics, and Cellular Reprogramming. Hypoxia or HIF-1,2 activation stimulates pluripotent gene expression and the Notch and Wnt signaling pathways, Changes in chromatin states also contribute to gene expression changes that lead to proliferation or self-renewal of stem cells.

The co-occupancy of transcription factors suggest the existence of a relationship between hypoxia and epigenetics when regulating cellular reprogramming (Figure 3). Several studies have studied this relationship. One example is the role of hypoxia in Epithelial-mesenchymal transition (EMT). EMT is the process that occurs when epithelial cells undergo reprogramming to become mesenchymal stem cells (Larue and Bellacosa, 2005). Mesenchymal stem cells are multipotent and play crucial roles in early embryonic development and the development of organs. Under hypoxia, promoters of epithelial genes e-cadherin and plakoglobin display decreased levels of H3K4 acetylation (H3K4ac) and increased levels of H3K27me3 (Wu et al., 2011). In contrast, promoters of mesenchymal genes N-cadherin and vimentin showed decreased levels of H3K27me3. In the reprogramming of cancer stem cells such as GSCs, hypoxia has shown to induce the activity of TET1 and TET3 (Prasad et al., 2017). TET1 and TET3 bind to Oct4 and Nanog regulatory regions and increase the activity of each transcription factor, thereby promoting the reprogramming of glioma cells to glioma stem cells.

Another promising area of research is the impact of hypoxia on the regulation of telomerase and the telomere. Telomeres are linear guanine-rich DNA structures at the ends of chromosomes, and their length determines the behaviors and life in many cells such as germ cells, cancer cells, and pluripotent stem cells. Telomeric DNA is synthesized by way of the ribonucleoprotein called telomerase, which contains a reverse transcriptase (TERT) subunit and an RNA component. Telomeres and TERT are essentially in iPSCs induction and maintenance (Teichroeb et al., 2016). TERT is highly conserved across species and ubiquitously present in pluripotent cells. Moreover, the TERT transcription can be enhanced by DNA methylation at the TERT-DMR via binding to nuclear lamina during cellular reprogramming (Takasawa et al., 2018). Hypoxia can promote telomerase TERT expression and HIF-1α mediates upregulation of TERT (Yu et al., 2006; Song et al., 2020). This has a significant impact on cellular reprogramming from somatic cells into pluripotent stem cells.

### Other Epigenetic Factors in Hypoxia

While the great majority of this review is focused on histone methylation, the potential role of histone acetylation and cellular reprogramming should not be ignored. An example of the impact of acetylation is the rapid acetylation that occurs after fertilization with the somatic nucleus. Before conception, the histones of mouse oocytes do not display any acetylation (Kim et al., 2003). However, if the same oocyte is implanted with a nucleus from a somatic cell, the histones become rapidly acetylated. This procedure is known as SCNT and through this process a permanently differentiated somatic cell undergoes cellular reprogramming to become a totipotent stem cell (Wilmut et al., 1997, 2002). This rapid acetylation that occurs after fertilization with the somatic nucleus indicates that acetylation plays a role in cellular reprogramming. More recently, a report suggests that reprogramming of H3K9ac is important for optimal SCNT efficiency and identifies Dux as a crucial transcription factor in this process (Yang et al., 2020). Despite the rapid acetylation, many of the fertilized oocytes reverted back to their previous unacetylated state 1–3 h after fertilization and the reprogramming is inefficient. In a study done by Rybouchkin et al. only 34% of the oocytes reached the blastocyst stage. However, when a histone decacetylase inhibitor (Trichostatin) was introduced during fertilization, a staggering 81% of the treated oocytes were able to reach the blastocyst stage compared to 41% of the non-treated oocytes (Rybouchkin et al., 2006). Another histone deacetylase inhibitor, valproic acid, found similar results (Huangfu et al., 2008). These studies indicate that inhibition of histone deacetylation can enhance cellular reprogramming.

Hypoxia has been proven to modulate histone acetylation by increasing histone acetylation at H3 and regulating the downstream processes of many genes. In addition, the initiation of hypoxia and induction of HIF-1α can increase the acetylation of H3 and H4 in neuroblastoma cells and increases the aggressiveness of neuroblastomas (Poljakova et al., 2014). A potential, exciting future area of research would be investigation to see if histone acetylation can play a role in cellular reprogramming of cancer cells to cancer stem cells.

## Looking Ahead

Hypoxia stimulation greatly affects gene expression and is considered to play an essential role in early embryo development, cell differentiation, and cellular reprogramming. Meanwhile, new techniques have been introduced to this field, such as the development of next-generation sequencing, single cell RNA sequencing, and micro-omics technologies. Recent studies have clearly depicted certain epigenetic changes, including DNA methylation, hydroxymethylation, histone modifications, organoids and 3D structure formation during early embryo development, iPSCs, and somatic cell reprogramming. There is potential for the use of the interplay of hypoxia and epigenetics in regenerative medicine. The existence of reprogrammed cells after hypoxia has been reported in multiple different types of tissue and organs (Shyh-Chang et al., 2013; Mosteiro et al., 2016). These conditional ischemic events via low oxygen stimulation are an intriguing prospect for future investigation. They suggest that a low oxygen environment may contribute or regulate the processes of tissue regeneration (Fang et al., 2018; Huels and Medema, 2018; Yui et al., 2018). There is undeniable involvement of HIF signaling and epigenetics during events that determine cell fate and cause rapid proliferation and differentiation. There is also evidence of epigenetic modifications at play in muscle regeneration and during differentiation of primary myogenic stem cells (Liu et al., 2013; Faralli et al., 2016). These factors and their association with tissue regeneration is potentially a new field for further study. Additionally, epigenetics have played a significant role in recent drug design (Harrison, 2012). Obviously, there will be a large benefit from the use of these epigenetic drugs or peptides to directly modulate the epigenome and normalize tissue regeneration.

## Author Contributions

NN drafted the manuscript and created the figures. XS discussed and edited the manuscript. RD revised and edited the manuscript. YL searched literature, structured, modified figures, and wrote and edited the manuscript. All author approved the final version of the manuscript.

## Conflict of Interest

YL was one of the executive editors for Journal of Cellular BioChemistry. The remaining authors declare that the research was conducted in the absence of any commercial or financial relationships that could be construed as a potential conflict of interest.
